# Integrated Immunological Engineering Platform for Scalable Production of iPSC‐Derived Megakaryocytes

**DOI:** 10.1111/tan.70870

**Published:** 2026-07-23

**Authors:** Rabea Dettmer, Alice Rovai, Linus Schröder, Thea Reinkens, Willem Wolkers, Rainer Blasczyk, Constanca Figueiredo

**Affiliations:** ^1^ Institute of Transfusion Medicine and Transplant Engineering, Hannover Medical School Hannover Germany; ^2^ Institute of Human Genetics, Hannover Medical School Hannover Germany; ^3^ Biostabilization Laboratory—Lower Saxony Centre for Biomedical Engineering, Implant Research and Development University of Veterinary Medicine Hannover Hannover Germany; ^4^ Unit for Reproductive Medicine—Clinic for Horses University of Veterinary Medicine Hannover Hannover Germany

**Keywords:** HLA silencing, iPSC‐derived megakaryocytes, megakaryocyte transfusion, transfusion medicine, universal cell therapy

## Abstract

Severe thrombocytopenia and impaired platelet function remain major clinical challenges in hematologic diseases, chemotherapy, bone marrow failure, surgery and obstetric haemorrhage. Conventional platelet transfusions are limited by donor dependency, short shelf life, storage constraints, and immune‐mediated platelet refractoriness caused primarily by HLA incompatibility. Here, we present a scalable, donor‐independent platform to generate universally compatible, low‐immunogenic megakaryocytes (MKs) from induced pluripotent stem cells (iPSCs). Human iPSCs were genetically engineered by shRNA‐mediated silencing of β2‐microglobulin (β2M) and/or class II transactivator (CIITA). Differentiation of HLA‐silenced iPSCs resulted in MKs with reduced HLA class I and II expression, capable to release functional platelets in vitro and sustain platelet production in humanised mice with physiological distribution. To enhance product safety, gamma irradiation effectively eliminated proliferative contaminants while preserving MK function. This platform integrates immune engineering with scalable iPSC‐based manufacturing and safety optimization, addressing key limitations of conventional donor‐derived platelet products. Together, these findings demonstrate that HLA‐silenced iPSC‐derived MKs represent a promising off‐the‐shelf source of functional platelets with reduced immunogenicity. By combining donor‐independent production, targeted immune engineering, and scalable manufacturing, this promising approach has the potential to improve transfusion support, overcome platelet refractoriness associated with HLA incompatibility, and expand access to readily available platelet therapies across diverse clinical settings.

**Abbreviations**
ADCC: antibody‐dependent cell‐mediated cytotoxicity
APEL: albumin polyvinyl alcohol essential lipids medium
β2M: β2‐Microglobulin
BMP4: bone morphogenetic protein 4
CIITA: class II transactivator
CNRQ: calibrated normalized relative quantity
DO: dissolved oxygen
GAPDH: glyceraldehyde‐3‐phosphate dehydrogenase
Gy: Gray
HLA: human leukocyte antigen
hu‐NSG: humanized nod.cg‐prkdc^scid il2rg^tm1wjl/szj mice
IFN‐γ: interferon gamma
IL: interleukin
IVIS: in vivo imaging system
MK: megakaryocyte
NanoLuc: NanoLuciferase
NT: non‐transduced
PBMC: peripheral blood mononuclear cell
PFHM: protein‐free hybridoma medium
PLT: platelet
ProA: proanthocyanidin A
ROCK: rho‐associated protein kinase
RT‐qPCR: reverse transcription quantitative polymerase chain reaction
SCF: stem cell factor
shRNA: short hairpin RNA
STAT: signal transducer and activator of transcription
TPO: thrombopoietin
VEGF: vascular endothelial growth factor

## 1 Introduction

Severe bleeding can occur in a wide range of clinical conditions and remains a major cause of morbidity and mortality. Over the past century, outcomes have improved substantially due to advances in transfusion medicine and optimised haemorrhage management [1, 2]. Early blood product support and effective bleeding control are strongly associated with improved survival. Platelet transfusions are a central component not only in the treatment of acute bleeding and trauma, but also in patients with hematologic disorders, chemotherapy‐ or radiation‐induced thrombocytopenia, bone marrow failure syndromes, major surgical procedures, obstetric haemorrhage and inherited or acquired platelet function defects [3, 4]. Despite their broad clinical relevance, the availability of platelet products remains limited by short shelf life, strict storage requirements, and reliance on donor supply [5, 6]. Globally, blood transfusions are among the most frequently performed medical interventions, with more than 110 million units administered annually, predominantly in high‐income countries [7]. Yet donor availability is increasingly strained by demographic changes, including population ageing and declining birth rates, which are expected to further reduce donation rates while clinical demand continues to rise [8]. Transfusion safety is simultaneously challenged by emerging pathogens and global disruptions such as the SARS‐CoV‐2 pandemic, which have demonstrated how rapidly blood supply chains, donor pools and clinical capacity can be destabilised. Platelet concentrates are particularly vulnerable because of their short shelf life, susceptibility to bacterial contamination, and storage‐related functional decline [9, 10]. Although platelet transfusions are essential for treating or preventing bleeding in thrombocytopenic patients, immune‐mediated platelet transfusion refractoriness, primarily driven by anti‐HLA antibodies, remains a major limitation, and access to HLA‐matched donors is often insufficient, especially for highly alloimmunized individuals [11, 12].

The in vitro generation of blood cells has emerged as a promising strategy to complement or replace donor‐derived transfusion products. Early preclinical studies have shown that CD34‐positive progenitor‐derived megakaryocytes (MKs) can safely release functional platelets in vivo [13, 14, 15]. Induced pluripotent stem cells (iPSCs) offer an essentially unlimited and ethically acceptable source for large‐scale MK production, and recent bioreactor technologies, including stirred‐suspension, microcarrier‐based and aggregate‐based systems, have advanced scalable manufacturing under serum‐free and xeno‐free conditions [16, 17, 18, 19]. Importantly, iPSC platforms enable precise genetic engineering to reduce immunogenicity, creating opportunities to overcome HLA‐driven rejection and platelet refractoriness. We have previously demonstrated that HLA silencing does not impair iPSC differentiation into MKs [20] and that gamma irradiation preserves MK functionality and platelet release [21], providing essential groundwork for developing HLA‐universal MK products. Given the central role of HLA molecules in immune recognition, the impact of HLA class I expression is well established, as it directly mediates recognition by cytotoxic T cells and contributes to platelet clearance [22, 23]. In contrast, the role of HLA class II, primarily known to mediate antigen presentation via antigen presenting cells and CD4^+^ T cells [24], remains less well defined in the context of platelet immunogenicity and alloimmune responses. Consideration of both HLA class I and class II may therefore be important for optimising the immunological compatibility of iPSC‐derived MK products.

Despite substantial progress in MK and platelet pharming, several critical gaps remain. Existing studies have rarely combined HLA‐immunomodulation, scalable manufacturing, detailed characterisation of non‐MK cell populations, in vivo assessment of platelet release, and irradiation‐based safety measures into a unified translational workflow. Moreover, the implications for clinical settings where universally compatible, shelf‐stable, and readily available hemostatic products are critically needed remain largely unexplored.

Building on these considerations, scalable production platforms are essential for translating iPSC‐derived MKs into clinically relevant products. Stirred‐suspension bioreactors offer a promising technological framework for generating transfusion‐relevant MK numbers under conditions compatible with industrial‐scale manufacturing. At the same time, comprehensive characterisation of co‐generated haematopoietic cell populations is increasingly recognised as a critical requirement for defining product identity, purity and safety, yet remains insufficiently addressed in most prior iPSC‐MK studies. Ensuring the inactivation of proliferative or undifferentiated contaminants is equally essential for clinical application [25, 26]. Gamma irradiation, already widely implemented in transfusion medicine, represents a viable strategy to enhance safety without compromising MK function and may therefore be integrated into future production workflows for iPSC‐derived MKs [27, 28].

Given the limitations of conventional platelet supply, where donor‐derived products may be scarce, short‐lived, or difficult to access, the development of universally compatible, donor‐independent MK products could significantly improve clinical availability. These unmet needs highlight the importance of combining immunological engineering, scalable production, thorough product characterisation and transfusion‐relevant safety measures. In this context, the present study aims to develop and validate such an approach as a foundation for next‐generation, off‐the‐shelf platelet and MK therapeutics suitable for a wide range of medical applications.

## 2 Methods and Materials

### 2.1 Peripheral Blood Mononuclear Cell (PBMC) Reprogramming

PBMCs were isolated from a healthy female donor with blood group O RhD‐negative. Reprogramming was performed using the CytoTune‐iPS 2.0 Sendai Reprogramming Kit (Thermo Fisher Scientific). The procedure followed the manufacturer's general guidance for Sendai virus–mediated delivery of reprogramming factors. Following transduction, cells were maintained under conditions supportive of pluripotency until emerging colonies displayed iPSC‐like morphology. One of the resulting reprogrammed iPSC lines was used for all experiments conducted in this study. The corresponding working cell bank stocks were established at passage 7, and all cells used in experiments were below passage 45, with most cultures at earlier passages.

### 2.2 iPSC Culture

iPSCs were cultured on cell‐culture vessels coated with human recombinant laminin‐521 (LN521; BioLamina). Cells were maintained in StemMACS iPS‐Brew medium (Miltenyi Biotec), which was refreshed daily. iPSCs were passaged approximately once per week. For routine passaging, ReLeSR (STEMCELL Technologies) was used to detach colonies in a controlled, non‐enzymatic manner, and cells were reseeded at dilution ratios typically ranging between 1:10 and 1:20, depending on colony density and morphology. For single‐cell dissociation, cultures were enzymatically detached using TrypLE Express (Life Technologies). After dissociation, cells were reseeded in medium supplemented with 10 μM ROCK inhibitor Y‐27632 (STEMCELL Technologies).

### 2.3 Immunofluorescence Staining

Immunofluorescence staining was performed according to standard procedures using the primary antibodies anti‐Oct3/4‐PE (BioLegend), anti‐Nanog‐AlexaFluor488 (BioLegend), anti‐CXCR4‐PE (Miltenyi), anti‐Tubb3‐AlexaFluor488 (Thermo Fisher Scientific), anti‐Pax6‐PE (Miltenyi), anti‐Tbx6 (Thermo Fisher Scientific) and anti‐Tbxt (Thermo Fisher Scientific).

### 2.4 Flow Cytometry

To assess differentiation efficiency, cells were stained with megakaryocyte‐specific antibodies anti‐CD41‐FITC (BioLegend), anti‐CD42a‐PE (BD Biosciences) and anti‐CD61‐APC (BioLegend). Cell viability was determined using Zombie Violet live/dead staining (BioLegend). Flow cytometric analyses were performed on a FACS Canto II system (BD Biosciences), and data were analysed using FlowJo software v10.8.1 (BD Biosciences). Absolute cell numbers were determined by flow cytometry using Cell Counting Beads (BioLegend) according to the manufacturer's instructions.

### 2.5 Karyotyping

Karyotype analysis was performed by the Institute of Human Genetics at Hannover Medical School. Adherent iPSCs were treated with colcemid (Invitrogen) to enrich cells in metaphase. The cells were then collected and processed according to established cytogenetic protocols for metaphase chromosome preparation. Fluorescence R‐banding was performed using chromomycin A3 and methyl green, as previously described [29]. For each clone, a minimum of 20 metaphase spreads with a resolution of at least 300 bands were analysed. Karyotypes were annotated according to the International System for Human Cytogenomic Nomenclature (ISCN). Karyotyping was performed at passage 12.

### 2.6 Tri‐Lineage Differentiation

Tri‐lineage differentiation was performed using the commercially available STEMdiff Tri‐lineage Differentiation Kit (STEMCELL Technologies) following the manufacturer's general guidelines. Differentiation efficiency toward the mesoderm, endoderm and ectoderm lineages was assessed by analyzing the expression of representative lineage‐specific markers. Marker expression was evaluated at the transcript level through gene expression analysis and at the protein level via immunofluorescence staining.

### 2.7 Gene Expression Analysis

For gene expression analysis, RNA was isolated using the Nucleospin RNA Plus Kit (Macherey‐Nagel) and reverse transcribed to cDNA using the high‐capacity cDNA reverse transcription kit (Applied Biosystems) according to the manufacturer's instructions. Transcript levels of β2M, CIITA, HLA‐DR were analysed using specific TaqMan Gene Expression Assays HS00984230_m1, Hs00932860_m1, Hs00219575_m1, Hs02758991_g1, respectively (Thermo Fisher Scientific). Transcript levels of pluripotency genes or lineage‐specific markers were measured using PowerSYBR Green PCR Master Mix (Thermo Fisher Scientific) and respective primer pairs (*POU5F1*: aatacctcagcctccagcagatg/tgcgtcacaccattgctattcttc, *SOX*: agctacagcatgatgcagga/ggtcatggagttgtactgca, *FOXA2*: ggaacaccactacgccttcaac/agtgcatcacctgttcgtaggc, *SOX17*: ccaagggcgagtcccgttatc/cacgacttgcccagcatcttg, *MIXL1*: ccgagtccaggatccaggta/ctctgacgccgagacttgg, *TBXT*: tgcttccctgagacccagtt/gatcacttctttcctttgcatcaag, *OTX2*: ggaagcactgtttgccaagacc/ctgttgttggcggcacttagct, *PAX6*: ctgaggaatcagagaagacaggc/atggagccagatgtgaaggagg). Technical triplicate reactions were carried out in StepOne real‐time PCR cycler (Thermo Fisher Scientific) and target gene levels were normalised to glyceraldehyde‐3‐phosphate dehydrogenase (GAPDH) using qBase plus (Biogazelle) and presented as relative gene expression termed CNRQ (calibrated normalized relative quantity).

### 2.8 Generation of HLA‐Universal iPSCs

Lentiviral vectors were designed to encode for intracellular NanoLuciferase as a reporter gene, β2M‐ or CIITA‐specific shRNAs to knockdown HLA class I and class II, respectively. A lentiviral vector encoding for a nonspecific shRNA was used as control. Lentiviral vector particles were generated in HEK‐293 T producer cells as previously described [30]. iPSCs were transduced overnight with the respective lentiviral vectors under standard culture conditions in the presence of protamine sulfate (Sigma‐Aldrich) to enhance transduction efficiency.

### 2.9 Megakaryocyte Differentiation

Differentiation was carried out in suspension, either at a small scale using 6‐well plates with 4 mL culture volume on an orbital shaker set to 90 rpm, or at a larger scale in a bioreactor with 120 mL culture volume, following a medium composition described in a previously established protocol [21]. For differentiation, iPSCs were seeded at 0.25 × 10^6^ cells/mL in StemMACS iPS‐Brew XF medium (Miltenyi Biotec) supplemented with 10 μM Y‐27632 dihydrochloride (Tocris Bioscience). On the following day (Day 0), differentiation was initiated by adding an equal volume of STEMdiff APEL2 medium (STEMCELL Technologies) supplemented with 100 ng/mL BMP4, 100 ng/mL VEGF (PeproTech), 5 μM CHIR99021 (STEMCELL Technologies) and 5% PFHMII (Gibco, Life Technologies). On Day 1, the medium was completely replaced with APEL2 containing 50 ng/mL BMP4, 50 ng/mL VEGF, and 5% PFHMII. Subsequent medium changes were performed on Days 4, 7 and 11 using STEMdiff APEL2 supplemented with 5% PFHMII, SCF, TPO (both 50 ng/mL), and IL‐3 (25 ng/mL; all cytokines from PeproTech). From Days 14, 18, 21 and 25, only SCF and TPO were added to the medium. For large‐scale megakaryocyte production, iPSCs were cultured in the DASbox Mini Bioreactor System (Eppendorf) under controlled stirring, aeration and temperature. Aeration was initiated at 21% O_2_ and 5% CO_2_ and subsequently regulated automatically by the system, while pH and dissolved oxygen (DO) levels were continuously monitored. DO levels were maintained above 30% by adjusting oxygen concentration and/or gas flow as required. Cells were stirred using an 8‐blade pitched impeller at 70 rpm, except on Day 0 (62 rpm). Shear stress was minimised by the addition of 1% P188 detergent (STEMCELL Technologies).

### 2.10 T Cell Proliferation Assay

MKs were sorted with anti‐CD61 magnetic beads (Miltenyi). T cells were first isolated from PBMCs using the Pan T Cell Isolation Kit (Miltenyi). Subsequently, the isolated T cells were labelled with the cell‐proliferation dye CPD eFluor 670 (Thermo Fisher Scientific). Co‐cultures were then established by seeding 2 × 10^4^ MKs together with the labelled T cells (in a ratio of 1:10) and 100 U IL‐2 (PreProTech) in triplicates in 250 μL RPMI 1640 (Gibco) supplemented with 5% human AB serum. The baseline time point of the incubation was determined by flow‐cytometric analysis. For this purpose, cells were stained with either Anti‐CD3‐FITC (BioLegend), Anti‐CD4‐PE/Cy7 (BioLegend), Anti‐CD8‐PE (BioLegend), or Anti‐CD3‐PerCP (BioLegend), Anti‐CD4‐FITC (BioLegend) and Anti‐CD8‐PE (BioLegend). After 6 days, cells were analysed again by flow cytometry using the same conjugated antibodies.

### 2.11 Antibody‐Dependent Cell‐Mediated Cytotoxicity Assay

The ADCC Reporter Bioassay Core Kit (Promega) was employed according to the manufacturer's general guidelines. MKs were sorted with anti‐CD61 magnetic beads (Miltenyi). Silenced and non‐silenced MKs were pretreated with interferon‐γ (100 ng/mL) for 24 h prior to analysis. On the assay day, MKs were exposed to unconjugated antibodies anti‐human HLA‐ABC or anti‐human HLA‐DR (both BioLegend), followed by co‐culture with the kit‐provided effector cells at a target‐to‐effector ratio of 1:6. After the defined incubation period, cell activation was quantified using the Bio‐Glo luciferase substrate, and luminescence was recorded with a Synergy 2 Multi‐Detection Microplate Reader (BioTek).

### 2.12 Animal Experiments

Humanised mice (humanized NOD.Cg‐Prkdc^scid Il2rg^tm1Wjl/SzJ; hu‐NSG; Jackson Laboratory) received an intravenous injection of 3 × 10^6^ MKs expressing the intracellular NanoLuciferase (NanoLuc) reporter. Blood samples were collected at 2, 24 h, 3 and 5 days post‐injection and were used to quantify the proportion of human platelets in the circulation. To assess the biodistribution of the transfused MKs, mice were administered up to 170 μL of Nano‐Glo Fluorofurimazine (Promega) In Vivo Substrate intraperitoneally under general anaesthesia at 24 h and 5 days following MK infusion. Subsequently, in vivo bioluminescence imaging was performed using an IVIS Lumina system to visualise and quantify NanoLuc‐positive cells.

### 2.13 Statistics and Experimental Procedures

Information on the number of independent biological replicates (*n*) is provided in the corresponding figure legends. Technical replicates were not included. Outliers were removed only when clear technical issues accounted for aberrant measurements and when individual values substantially deviated from the median or mean of the respective biological group. Statistical analyses were performed using unpaired two‐tailed Student’s t‐test for comparisons between two groups and one‐way ANOVA followed by Tukey’s post hoc test for multiple‐group comparisons. Statistical evaluation followed the conventional *p* value–based null hypothesis significance testing framework. Significance thresholds were defined as *p < 0.05, **p < 0.01, ***p < 0.001 and ****p < 0.0002. Results were described as significant only when the *p* value fell below the predefined α‐level (α < 0.05), leading to rejection of the null hypothesis. Statistical analyses were omitted when the question addressed was not relevant to the central conclusions of the study.

## 3 Results

### 3.1 Generation of a Low‐Immunogenic HLA‐Universal iPSC Line

The generation of low‐immunogenic, HLA‐universal iPSCs is of particular advantage for producing allogeneic MKs and platelets, as it minimises the risk of immune rejection and alloimmunization in transfusion settings. Platelets express a range of immunogenic surface molecules, including HLA antigens, which can lead to rapid clearance or refractoriness in sensitized recipients. Establishing an iPSC line with reduced immunogenicity therefore provides a robust and scalable source for producing universally compatible MK and PLT products. Furthermore, the use of iPSCs derived from a female donor is advantageous, as female cells do not express Y‐chromosome–encoded minor histocompatibility antigens such as HY antigens, which are known to elicit strong immune responses, particularly in female recipients. Moreover, selecting a donor with blood group O provides an additional benefit, as the absence of A and B antigens reduces the risk of ABO‐incompatible immune reactions and supports broad transfusion compatibility.

**FIGURE 1:**
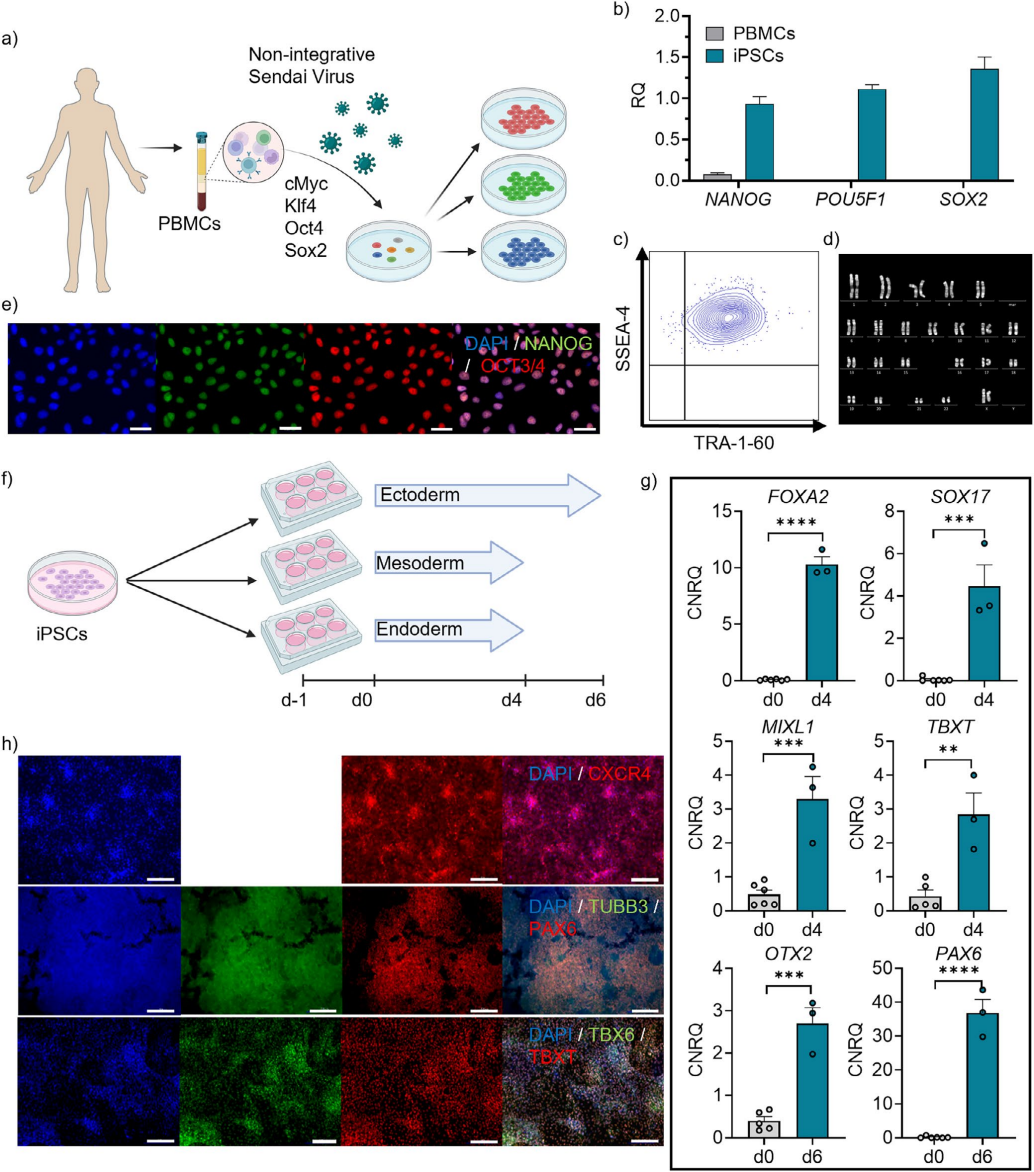
Derivation and Tri‐lineage Differentiation of Female blood group O and Rhesus negative iPSCs. (a) Schematic overview of the reprogramming protocol using Sendai viruses. The red, green, and blue cell colonies represent independent colonies originating from single‐cell clones after reprogramming. (b) Gene expression analysis (relative quantity, RQ) of the endogenous pluripotency markers NANOG, POU5F1 and SOX2 in the selected reprogrammed iPSC clone relative to the parental PBMC sample from the same donor. RT‐qPCR measurements were performed in technical triplicates, and data are presented as mean ± SD. (c) Flow cytometric assessment of pluripotency marker expression (SSEA‐4 and TRA‐1‐60) in the derived iPSCs. Flow cytometric analysis was performed by acquiring a total of 10,000 events. (d) Representative karyogram of the established iPSC line. (e) Immunofluorescence staining for pluripotency markers NANOG (green) and OCT3/4 (red). Scale bar = 50 μm. (f) Schematic overview of trilineage differentiation using the STEMdiff Trilineage Differentiation Kit (see Methods) (g) Gene expression analysis (calibrated normalized relative quantity, CNRQ) of lineage‐specific markers following differentiation into mesoderm (Day 4: *MIXL1*, *TBXT*), endoderm (Day 4: *FOXA2*, *SOX17*) and ectoderm (Day 6: *OTX2*, *PAX6*), relative to undifferentiated iPSCs (Day 0). Data represent mean ± SEM of 3–5 independent experiments performed using cells from different passages. (h) Immunofluorescence detection of representative lineage‐specific markers for endoderm (CXCR4, red), ectoderm (TUBB3, green; PAX6, red) and mesoderm (TBX6, green; TBXT, red). Scale bar = 200 μm. Significance thresholds were defined as * *p* < 0.05, **p < 0.01, ***p < 0.001 and ****p < 0.0002.

Reprogramming of PBMCs obtained from a female donor with blood group O negative (Figure 1a) generated an iPSC line that robustly expressed the pluripotency markers NANOG, POU5F1, and in which nearly all cells expressed the pluripotency surface markers SSEA‐4 and TRA‐1‐60 (Figure 1b,c,e). The generated iPSC line exhibited a normal karyotype (Figure 1d) and demonstrated the capacity to differentiate into all three germ layers following tri‐lineage differentiation (Figure 1f). Tri‐lineage differentiation potential was confirmed by the expression of germ layer–specific markers, including CXCR4, FOXA2 and SOX17 for endoderm, TUBB3, PAX6 and OTX2 for ectoderm and TBX6, TBXT and MIXL1 for mesoderm, as assessed by RT‐qPCR analysis and/or protein‐level detection via immunofluorescence staining (Figure 1g,h).

**FIGURE 2:**
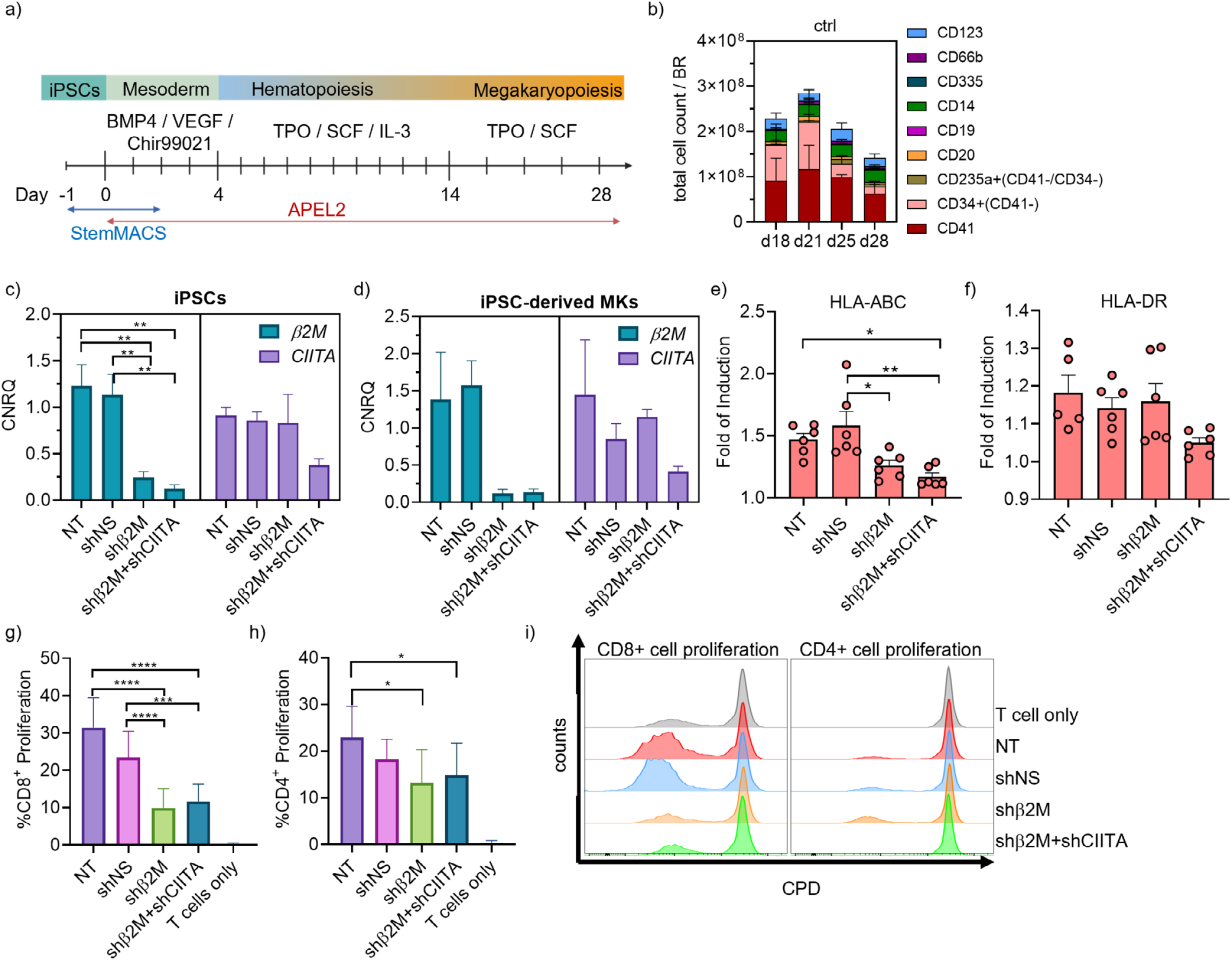
Assessment of Immunogenicity of HLA‐Universal iPSC‐Derived Megakaryocytes via ADCC and T Cell Proliferation Assays. (a) Representative schematic of the MK differentiation protocol. (b) Characterisation of the total cell population generated during differentiation, including haematopoietic byproducts. Data represent mean ± SEM of 3–4 independent experiments performed using cells from different passages. (c) Gene expression analysis (CNRQ) of β*2M* and *CIITA* in the generated iPSCs following β2M or β2M/CIITA silencing compared to non‐transduced (NT) controls and cells transduced with a non‐targeting shRNA (shNS). Data represent mean ± SEM using cells of 3 separate passages. (d) Gene expression analysis (CNRQ) of *β2M* and *CIITA* in iPSC‐derived MKs following *β2M* or *β2M/CIITA* silencing compared to non‐transduced (NT) controls and cells transduced with a non‐targeting shRNA (shNS). Data represent mean ± SEM from 3 independent differentiations. (e) ADCC assay showing fold induction of effector cell activation after incubation with anti‐HLA‐ABC antibody and (f) anti‐HLA‐DR antibody. Data represent mean ± SEM of 5–6 independent experiments performed with cells derived from separate passages. (g) CD8^+^ and (h) CD4^+^ T cell proliferation after co‐culture with β2M‐ or β2M/CIITA‐silenced MKs compared to non‐transduced (NT) controls and cells transduced with non‐targeting shRNA (shNS). *n* = 11, Data represent mean ± SEM of 11 independent experiments performed with 11 different T cell donors. (i) Representative flow cytometry analysis of T cell proliferation. Significance thresholds were defined as *p < 0.05, **p < 0.01, ***p < 0.001 and ****p < 0.0002.

The newly generated iPSC line was efficiently transduced with lentiviral vectors encoding short hairpin RNAs (shRNAs) targeting β2‐microglobulin (β2M) and/or class II transactivator (CIITA). β2M is a critical component of HLA class I molecules, required for their proper assembly and surface expression, while CIITA is the master transcriptional regulator of HLA class II gene expression. Knockdown of β2M was highly effective, with expression levels significantly reduced in both β2M‐silenced and β2M/CIITA double‐silenced iPSCs compared to non‐transduced (NT) control (79.9% reduction of expression for β2M‐silenced and 89.9% reduction for β2M/CIITA double‐silenced iPSCs) and cells transduced with a non‐targeting shRNA (shNS) (Figure 2c). Similarly, CIITA transcript levels were substantially decreased in the β2M/CIITA double‐silenced iPSCs relative to both control groups (52.2% reduction of expression compared to NT) (Figure 2c), demonstrating robust and specific silencing of the target genes. Importantly, efficient gene silencing was maintained following differentiation into MKs, with β2M expression reduced by approximately 91.5% in shβ2M MKs and 90.3% in shβ2M/CIITA double silenced MKs, while CIITA expression remained reduced by approximately 71.7% in shβ2M/CIITA double silenced MKs compared to NT controls (Figure 2d).

In summary, we established low‐immunogenic, HLA‐universal iPSC lines exhibiting full pluripotency and efficient, targeted suppression of β2M and/or CIITA, providing a solid foundation for the production of immunologically compatible MKs and platelets.

### 3.2 Knocking Down HLA Expression Reduces Immunogenicity of iPSC‐Derived MKs

MKs were generated from the respective iPSC lines using a previously established differentiation protocol as depicted in Figure 2a. In vitro assays demonstrated that MKs derived from β2M‐silenced and β2M/CIITA double‐silenced iPSCs exhibited reduced effector cell activation in a simulated ADCC assay with an anti‐HLA‐ABC antibody (targeting HLA class I). Relative to the non‐transduced (NT) control, overall fold‐induction values were decreased by 14.28% for β2M‐silenced MKs and 20.60% for β2M/CIITA double‐silenced MKs (*p* < 0.05; Figure 2e). As fold induction was normalised to the negative control (baseline = 1), these reductions correspond to an approximately 44.6% and 64.3% decrease, respectively, in the induced effector cell response above baseline. In contrast, assays using an anti‐HLA‐DR antibody (targeting HLA class II) showed only a minor, non‐significant reduction in overall fold‐induction values for β2M/CIITA‐silenced MKs (11.17%), consistent with the generally low baseline activity of effector cells against HLA class II in this system (Figure 2f). When considering the induced response above baseline, this corresponds to a reduction of approximately 72.5%; however, given the low magnitude of HLA‐II‐mediated effector activation and the lack of statistical significance, these findings suggest only limited functional relevance in this assay setting. When co‐cultured with T cells, MKs derived from β2M‐ and β2M/CIITA‐silenced iPSCs induced significantly lower proliferation of CD8^+^ T cells (9.93% ± 5.16% and 11.68% ± 4.61%, *p* < 0.0002) compared to controls (31.42% ± 8.05% and 23.41% ± 7.07%) (Figure 2g,i). CD4^+^ T cell proliferation was also significantly reduced (13.22% ± 7.11% and 14.89% ± 6.78%, *p* < 0.05) relative to the non‐transduced (NT) control (22.91% ± 6.75%) (Figure 2h,i). However, no significant difference in T cell proliferation was observed between MKs derived from β2M‐silenced and β2M/CIITA double‐silenced iPSCs.

Together, these results confirm that targeted silencing of β2M in MKs effectively diminishes HLA class I‐mediated immune recognition, reducing both ADCC responses and CD8^+^ T cell proliferation, whereas CIITA knockdown alone has a limited impact on HLA class II‐mediated effector responses under the tested conditions.

### 3.3 Generation and Characterisation of iPSC‐Derived MKs

The production of MKs was carried out in a bioreactor system, providing a controlled and standardised environment for differentiation. MK differentiation was performed following a previously established protocol (Figure 2a). During differentiation into MKs, additional haematopoietic cell types naturally emerge. For clinical application, it is essential to define which contaminating cell populations are present in the final product and to establish threshold values that ensure the safety of the transfused cell preparation. We therefore characterised the contaminating populations using markers for distinct haematopoietic lineages (Figure 2b). In addition to MKs (40.6% ± 7.8% CD41^+^), the cultures predominantly contained monocytes and progenitor cells (13.1% ± 4.8% CD14^+^, 19.1% ± 12.9% CD34^+^), as well as a minor fraction of erythroid cells (1.8% ± 0.5% CD235a^+^). Immune cells were present only at very low frequencies (0.15% ± 0.17% CD19^+^, 2.0% ± 1.0% CD66b^+^, 0.7% ± 0.2% CD335^+^), and T cells were entirely absent (0% CD3^+^/CD4^+^, 0% CD3^+^/CD8^+^), thereby minimising the risk of immunological adverse effects. These results demonstrate that the bioreactor‐based differentiation reliably produces MKs with a defined cellular composition suitable for further preclinical and therapeutic applications.

### 3.4 Sustained Platelet Release From Transfused iPSC‐Derived MKs

**FIGURE 3:**
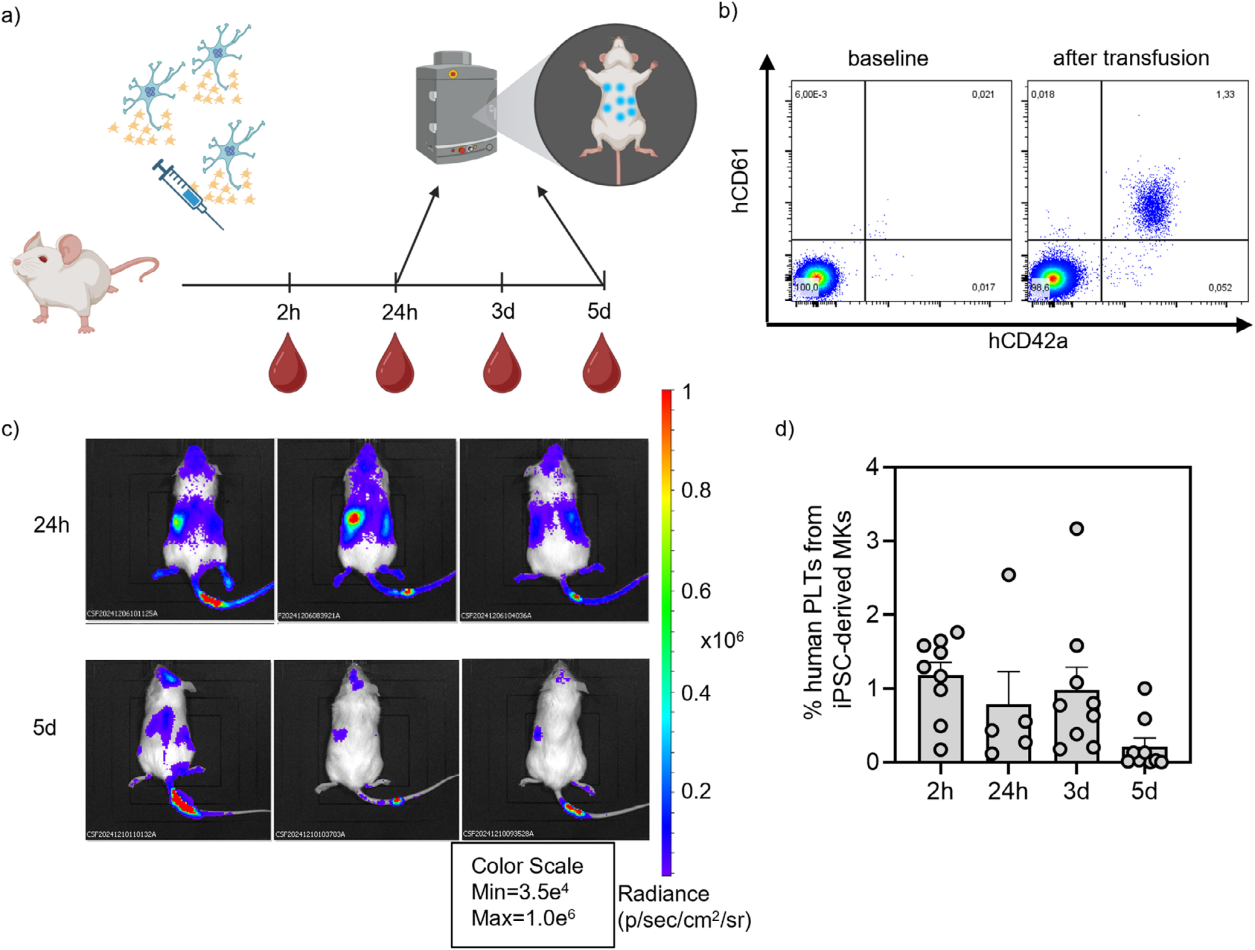
In Vivo Platelet Release and Biodistribution of Transfused iPSC‐Derived Megakaryocytes. (a) Schematic overview of the animal experimental setup. (b) Representative flow cytometric analysis of human platelet markers (hCD61 and hCD42a) in the peripheral blood of transfused mice. (c) Biodistribution of NanoLuc‐labelled cells in transfused mice at 24 h and 5 days after transfusion. Representative bioluminescence images of three individual mice are shown. (d) Percentage of human platelets derived from the 3 × 10^6^ transfused MKs in the total PLT population (mouse + human PLTs) in the circulation, determined by flow cytometry at 2, 24 h, 3 and 5 days following transfusion. Data represent mean ± SEM and were obtained from 5 to 9 mice transfused with iPSC‐derived MKs.

To evaluate the in vivo functionality of the generated MKs, iPSC‐derived MKs expressing an intracellular NanoLuc reporter were transfused into humanized NSG mice (Figure 3a). This approach enabled simultaneous monitoring of platelet release into the circulation and biodistribution of the infused MKs over time. The experiment was designed to assess the persistence of human platelet production and organ‐specific distribution of the administered cells. Following transfusion of the iPSC‐derived MKs, human platelets were detectable in the peripheral blood of recipient mice for at least 5 days, demonstrating sustained in vivo platelet release (Figure 3b,d). Bioluminescence imaging performed 24 h after MK infusion revealed a homogeneous distribution of reporter‐positive cells throughout the organism (Figure 3c). Notably, no abnormal or excessive accumulation was observed in the lung tissue. Instead, a physiological enrichment was detected in the spleen, consistent with its known role in mice as a natural reservoir that stores approximately 10%–30% of circulating platelets. Together, these findings demonstrate successful PLT release from MKs in the blood circulation in vivo and functional contribution of the transfused MKs in the murine model.

### 3.5 Irradiation to Enhance Safety of Transfused iPSC‐Derived MKs

**FIGURE 4:**
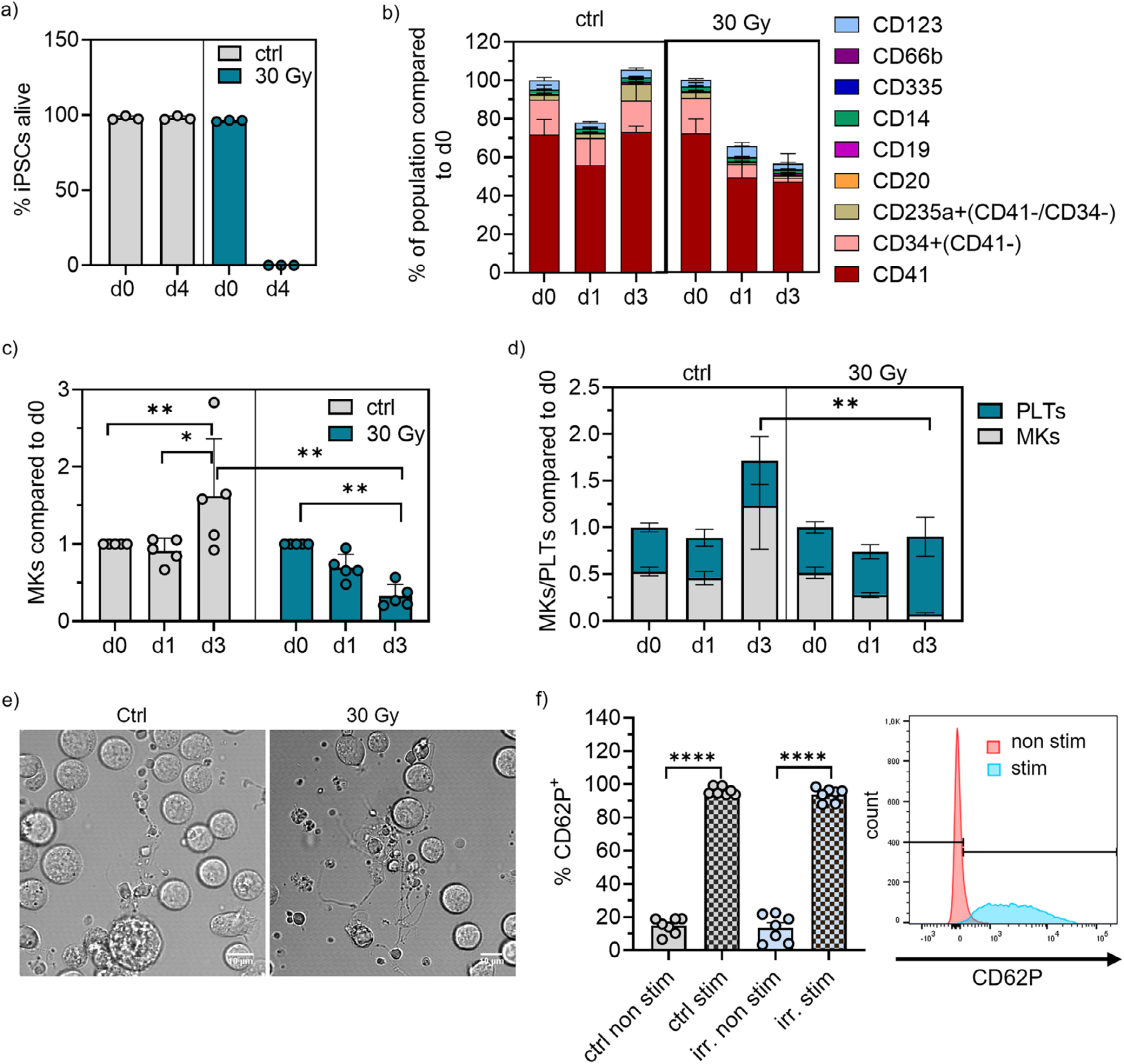
Safety Assessment of the Gamma‐Irradiated MK Cell Product. (a) Viability of iPSCs on Day 0 and Day 4 following irradiation with 30 Gy or without irradiation (ctrl). Cell viability was assessed by live/dead staining using Zombie Violet Fixable Viability Dye. Data represent mean ± SEM of 3 independent experiments performed using cells derived from separate passages. (b) Percentage of haematopoietic cells within the total cell population generated during MK differentiation on Day 0, Day 1 and Day 3 after 30 Gy irradiation or without irradiation (ctrl). Data represent mean ± SEM of 3 independent experiments with cells obtained from separate passages. (c) Viable MKs quantified on Day 0, Day 1 and Day 3 after 30 Gy irradiation or without irradiation (ctrl), normalized to Day 0. Data represent mean ± SEM of 5 independent experiments performed using cells derived from separate passages. (d) Quantification of MKs and PLTs on Day 0, Day 1 and Day 3 after irradiation or without irradiation (ctrl), normalized to Day 0. Data represent mean ± SEM of 3 independent experiments of cells derived from separate passages. (e) Representative microscopic images of proplatelet‐forming MKs on Day 1 after 30 Gy irradiation or without irradiation (ctrl). Scale bar = 10 μm. (f) Flow cytometric analysis comparing the platelet activation marker CD62P of non‐stimulated (non stim) and stimulated (stim) PLTs derived from non‐irradiated iPSC‐derived MKs (ctrl) and from megakaryocytes exposed to 30 Gy irradiation (irr). Data represent mean ± SEM of 5 independent experiments of cells derived from separate passages. Significance thresholds were defined as *p < 0.05, **p < 0.01, ***p < 0.001 and ****p < 0.0002.

In clinical transfusion settings, ensuring the safety of iPSC‐derived cell products requires strategies to prevent engraftment and eliminate the risk of teratoma formation from residual iPSCs. Gamma irradiation is commonly employed to halt the proliferation of potentially contaminating cells while maintaining the functionality of differentiated cell populations. To evaluate this approach, contaminating cells were subjected to a single gamma irradiation of 30 Gray (Gy). Post‐irradiation, iPSCs did not survive (Figure 4a), whereas MKs retained full capacity to form proplatelets (Figure 4e) and release platelets (Figure 4d). Released PLTs were functional, and irradiation did not impair their ability to become activated, as demonstrated by the expression of the activation marker CD62P following stimulation with the agonists thrombin and ADP. Expression of CD62P in non‐stimulated platelets showed no significant difference between non‐irradiated and irradiated conditions (14.1% ± 6.6% vs. 14.4% ± 8.1%, respectively). Similarly, no difference was observed in stimulated platelets (95.7% ± 2.5% non‐irradiated vs. 94.8% ± 4.0% irradiated) (Figure 4f). Notably, in both conditions CD62P expression increased significantly from non‐stimulated to stimulated platelets (*p* < 0.0002), reaching levels above 90% following stimulation. Irradiation resulted in a reduction of MK numbers, while platelet release increased, indicating that MKs ceased proliferation but continued to mature and release platelets (Figure 4d). In contrast, non‐irradiated controls showed an increase in MK numbers (162% ± 66% relative to Day 0). Furthermore, irradiation caused a marked decrease in the proportion of CD41^−^ contaminating cells, dropping to 5.9% ± 3.3% after 3 days, compared to 28.6% ± 8.18% at Day 0. Non‐irradiated controls displayed ongoing proliferation and differentiation, with contaminating cells remaining largely stable (22.2% ± 22.7% on Day 3 vs. 28.8% ± 14.9% on Day 0) (Figure 4b). These results demonstrate that gamma irradiation effectively eliminates proliferative contaminants while preserving the functional platelet‐producing capacity of iPSC‐derived MKs. Together, these findings support gamma irradiation as an effective safety measure to prevent contaminating cell proliferation while preserving the platelet‐producing function of iPSC‐derived MKs for potential clinical transfusion applications.

## 4 Discussion

Platelet transfusions remain a cornerstone of supportive care in patients with thrombocytopenia, yet their clinical use is limited by donor dependency, immunogenicity and logistical constraints [31, 32]. Developing a universally compatible, off‐the‐shelf MK product from iPSCs could overcome these challenges and provide a scalable, readily available alternative for a wide range of patients.

In this study, we established the generation of low‐immunogenic iPSCs derived from a blood group O and Rhesus negative female donor. While these iPSCs demonstrated robust pluripotency, genomic stability, and trilineage differentiation capacity, our primary aim was to evaluate the feasibility of developing a broadly compatible, hypoimmunogenic platform for clinical MK production. Targeted HLA silencing via shRNA‐mediated knockdown of β2M and CIITA yielded MKs with markedly reduced immune recognition, as evidenced by diminished effector cell activation in ADCC assays and reduced CD8+ and CD4+ T cell proliferation. Our findings suggest that this effect is largely driven by HLA class I downregulation, as β2M silencing alone was sufficient to significantly reduce ADCC responses and CD8^+^ T cell proliferation. In contrast, additional CIITA‐mediated suppression of HLA class II did not further attenuate immune responses, and only modest, non‐significant effects were observed. These observations are consistent with the predominant role of HLA class I in mediating platelet and MK immunogenicity [12], while also indicating that HLA class II may play a more limited or context‐dependent role in this setting. Partial HLA silencing rather than complete knockout may confer additional benefits by mitigating NK cell‐mediated cytotoxicity triggered by complete absence of HLA molecules, a phenomenon described as the “missing self” response. Previous studies have highlighted that hypoimmunogenic iPSCs lacking HLA class I are vulnerable to NK cell attack, whereas residual HLA expression or incorporation of non‐classical HLA molecules, such as HLA‐E or HLA‐G, can provide protection against innate immunity [33, 34]. Importantly, the partial reduction in effector cell activation observed in ADCC assays is consistent with the intended strategy of controlled HLA modulation, achieving reduced immune recognition while maintaining sufficient HLA class I expression to preserve inhibitory signalling and limit NK cell‐mediated “missing self” recognition. Future studies will be required to determine whether this degree of immune attenuation is sufficient to support clinically relevant platelet persistence and function.

Comprehensive characterisation of non‐MK populations generated during differentiation allowed precise definition of the final product's cellular composition. This is particularly important because additional hematopoietic lineages are consistently produced alongside MKs in iPSC‐based differentiation systems [35, 36]. Understanding their identity, abundance, and immunological profile supports improved process control, provides important quality attributes for regulatory assessment, and increases confidence in product safety by confirming minimal immune cell contamination. Collectively, these results demonstrate the feasibility of producing iPSC‐derived MKs at clinically relevant scale with a well‐defined cellular composition, consistent functional properties, and low levels of contaminating immune cells, thereby strengthening the translational potential of this manufacturing strategy.

These data provide an initial validation of a scalable manufacturing concept to produce universally compatible, low‐immunogenic MKs as an off‐the‐shelf resource, independent of HLA matching or ABO typing. Such a platform could simplify logistics and broaden patient access, particularly in settings requiring rapid availability of safe platelet products. It may also prevent patient alloimmunization and help address platelet transfusion refractoriness in already sensitised patients. This is a major clinical challenge for patients who develop alloimmune responses after repeated transfusions and require difficult‐to‐source HLA‐matched products [11, 37]. By enabling the production of virtually unlimited numbers of low‐immunogenic MKs and platelets suitable for any patient, this strategy could greatly improve the consistent availability of cellular products. By demonstrating that HLA‐silenced iPSC‐derived MKs retain safety‐relevant characteristics while exhibiting reduced immunogenicity, this work lays a conceptual foundation for universally applicable, reliable, and accessible MKs for diverse clinical settings worldwide.

Transfusion experiments in humanized NSG mice confirmed that iPSC‐derived MKs produced circulating human platelets for at least 5 days, with physiological biodistribution and no detectable abnormal pulmonary accumulation at the time points analysed. These findings demonstrate that in vitro generated MKs are capable of in vivo platelet release, supporting their functional relevance as a transfusion product. Interpretation of these data requires consideration of species‐specific differences in MK trafficking and clearance. Several studies have reported that the lung serves as a major site for MK retention, fragmentation, and platelet production in mice, resulting in variable transient pulmonary accumulation depending on injection route, cell dose and experimental design [38, 39, 40]. In contrast, our data did not reveal pronounced long‐term retention, suggesting either rapid processing of MKs in the pulmonary microvasculature or accelerated clearance. An important factor contributing to reduced persistence of human MKs in NSG mice is the activity of the murine mononuclear phagocyte system. Although NSG mice lack functional T, B and NK cells, macrophage populations remain partially active and are known to mediate xenogeneic cell clearance [41, 42, 43]. The absence of macrophage depletion in our model therefore likely results in earlier removal of transfused human MKs than would be expected in a fully homologous human context. In humans, species‐matched recognition pathways, differences in phagocytic receptor compatibility, and the larger pulmonary capillary network may support longer residence time and more efficient in vivo maturation and platelet release. These findings indicate that the mouse model provides a conservative estimate of MK persistence, demonstrating functionality but not necessarily reflecting the full therapeutic window achievable in humans. Future studies using models with reduced phagocytic activity, extended follow‐up time points, or advanced in vivo imaging will be important to more accurately define MK processing and survival. Nevertheless, the ability of HLA‐universal MKs to generate physiologically distributed human platelets in vivo supports their clinical relevance and highlights the potential to reduce alloimmune complications such as alloimmunization and platelet refractoriness associated with conventional transfusions.

To enhance product safety, gamma irradiation was applied to eliminate residual iPSCs and proliferative contaminants, substantially reducing the proportion of CD41 negative cells. This approach aligns with established clinical practice, as irradiated platelet concentrates are routinely used to prevent transfusion‐associated graft‐versus‐host disease, demonstrating that irradiation is a clinically compatible and regulatory familiar strategy for iPSC‐derived products [25, 44, 45]. Importantly, irradiation of MKs did not impair the functional properties of the released platelets. iPSC‐PLTs retained their ability to become activated upon stimulation, as demonstrated by the expression of the activation marker CD62P following agonist stimulation. CD62P is translocated from α‐granules to the platelet surface during activation and serves as a well‐established indicator of platelet degranulation and functional responsiveness [46]. These findings suggest that irradiation effectively improves product safety without compromising the activation capacity of the resulting platelets.

Despite the encouraging results, limitations remain. Humanised mice provide important proof of concept data, but their innate immune system, stromal interactions and macrophage‐driven clearance differ from humans and may underestimate MK survival [42, 47]. Additionally, although HLA‐universal iPSCs address major alloantigen disparities, residual minor antigens, including blood group and minor histocompatibility antigens, may still elicit immune responses in a subset of highly sensitised patients [48]. Consequently, in our study design, we utilise female donors with blood group O, Rhesus negative, in order to minimize potential immunogenicity and reduce the likelihood of residual antigen‐mediated immune responses.

Overall, these considerations underscore the strong translational potential of universal iPSC‐derived MK products. In clinical situations where conventional donor‐derived platelet supplies are limited or in high demand, availability can be restricted by short shelf life, storage requirements, and the need for HLA compatibility testing [49, 50]. The platform established in this study, combining targeted HLA‐knockdown, scalable differentiation methods, and gamma irradiation for safety, addresses these challenges by enabling the generation of off‐the‐shelf MKs capable of producing functional platelets independent of donor availability. Such a capability could significantly improve transfusion support for patients undergoing major surgery, chemotherapy, bone marrow transplantation, or other medical conditions associated with thrombocytopenia and bleeding. In this context, the ability to produce universally compatible, low‐immunogenic MKs at scale represents an important advance toward readily available, off‐the‐shelf platelet therapies with broad applicability across hospitals and healthcare systems.

## Author Contributions

R.D. and C.F. contributed to the study concept and design, methodology, data analysis, investigation, validation and drafting of the manuscript. A.R. contributed to the study concept and design, methodology, and data analysis. C.F. and R.B. were responsible for funding acquisition. W.W. and R.B. provided scientific advice and contributed to the review and editing of the manuscript. L.S. and T.R. contributed to data analysis. All authors have read and approved the final version of the manuscript.

## Funding

This work was supported by the Bundesamt für Ausrüstung, Informationstechnik und Nutzung der Bundeswehr, BAAINBw MHH‐HF‐ZB3‐2024 and the Stiftung des DRK‐Blutspendienst NSTOB StiBSD/2023/D3745.

## Ethics Statement

This study was approved by the Ethics Committee of the Medizinische Hochschule Hannover. Written informed consent was obtained from all donors prior to the derivation of induced pluripotent stem cells, in accordance with relevant guidelines and regulations. All animal experiments were conducted in compliance with applicable national and institutional guidelines and were authorized by the competent authority.

## Conflicts of Interest

The authors declare no conflicts of interest.

## Data Availability

The data that support the findings of this study are available from the corresponding author upon reasonable request. The iPSC‐line generated in this study is available upon reasonable request from the corresponding author.
